# A Retinoic Acid Responsive *Hoxa3* Transgene Expressed in Embryonic Pharyngeal Endoderm, Cardiac Neural Crest and a Subdomain of the Second Heart Field

**DOI:** 10.1371/journal.pone.0027624

**Published:** 2011-11-16

**Authors:** Nata Y. S.-G. Diman, Sophie Remacle, Nicolas Bertrand, Jacques J. Picard, Stéphane Zaffran, René Rezsohazy

**Affiliations:** 1 Molecular and Cellular Animal Embryology group, Life Sciences Institute, Université catholique de Louvain, Louvain-la-Neuve, Belgium; 2 UMR910, Aix-Marseille University, Marseille, France; 3 Medical Genetics and Functional Genomics, Inserm UMR_S910, Marseille, France; 4 Faculty of Medicine, Université catholique de Louvain, Brussels, Belgium; Brigham and Women's Hospital, United States of America

## Abstract

A transgenic mouse line harbouring a β-galacdosidase reporter gene controlled by the proximal 2 kb promoter of *Hoxa3* was previously generated to investigate the regulatory cues governing *Hoxa3* expression in the mouse. Examination of transgenic embryos from embryonic day (E) 8.0 to E15.5 revealed regionally restricted reporter activity in the developing heart. Indeed, transgene expression specifically delineated cells from three distinct lineages: a subpopulation of the second heart field contributing to outflow tract myocardium, the cardiac neural crest cells and the pharyngeal endoderm. Manipulation of the Retinoic Acid (RA) signaling pathway showed that RA is required for correct expression of the transgene. Therefore, this transgenic line may serve as a cardiosensor line of particular interest for further analysis of outflow tract development.

## Introduction

Cardiac development requires specification, proliferation, migration and differentiation of progenitor cells from diverse tissues of the embryo [1]. Myocardial progenitor cells in the anterior splanchnic mesoderm are destined to form the left ventricle and contribute to the formation of the atrio-ventricular canal and the atria. These cells characterize the first heart field (FHF) as opposed to a population of cardiac progenitor cells defining the second heart field (SHF) [2], [3]. SHF proliferating progenitor cells are located in the pharyngeal mesoderm lying medial to the FHF. Initially, FHF cells, which differentiate at embryonic day (E)7.5, form the primitive heart tube while subsequent addition of SHF cells at both anterior and posterior poles lead to elongation and looping of the forming heart and contribute to right ventricular, outflow tract (OFT) and atrial myocardium [4], [5], [6], [7]. SHF cells express genes including *Fgf8*, *Foxh1*, *Tbx1*, *Isl1* and *Nkx2.5* of which inactivation leads to defects in the development of SHF progenitors and subsequently of the OFT [5], [8], [9], [10], [11].

Neural crest cells are multipotent stem cells that originate from the dorsal neural tube and give rise to various structures such as nerves, ganglia, cartilages, bones and connective tissue [12], [13]. Cardiac neural crest cells are a subdivision of the cranial crest originating from the level of the otic placode to the caudal border of somite 3, corresponding to rhombomeres 6, 7 and 8 [12], [14], [15]. Cells of the neural crest migrate to the third, fourth and sixth pharyngeal arches (PA), where they are largely devoted to glandular and vascular development. Cardiac neural crest cells play an important role in patterning the aortic arch arteries and form the smooth muscle tunics of the great arteries. The migration patterns of neural crest cells in mammalian species have been identified by fate-mapping studies with gene expression markers for neural crest cells [16], [17], [18]. Thus, a subset of the cardiac neural crest cells migrates between the aortic sac and the pharyngeal endoderm and infiltrates the cardiac outflow cushions [16], [17], [18]. Ablation and quail-chick chimera experiments showed that cardiac neural crest cells are absolutely required to form the aorticopulmonary septum dividing the cardiac arterial pole into systemic and pulmonary circulations [12].

The morphogenesis of the arterial pole (outflow tract) of the heart is a complex process that is defective in many congenital heart defects and depends on the interaction between cardiac neural crest and SHF cells after formation of the primitive heart tube [12], [19], [20]. Indeed, addition of SHF derived cells and migration of cardiac neural crest cells into the OFT temporally overlap (embryonic days 9.5–10.5) [4], [21]. Recent data have suggested that a cross-talk between these two cell populations is crucial for normal OFT development [22], [23]. First, ablation of cardiac neural crest results in failure of the OFT to lengthen by addition of myocardial progenitors from the SHF [19], [20]. Second, loss of *Tbx1* in pharyngeal mesoderm (SHF) can negatively impact on cardiac neural crest cells [11]. Pharyngeal endoderm has been implicated as a third player in development of the OFT. Indeed, a study showed that Sonic Hedgehog produced by pharyngeal endodermal cells has a direct or indirect action on cardiac neural crest and SHF cells survival, respectively [22]. These data suggest that normal OFT morphogenesis depends on an intricate interplay between cardiac neural crest, SHF and pharyngeal endoderm.

*Hox* genes encode a class of transcription factors that play an important role in patterning vertebrate axial development [24], [25]. For example, the axial identity of the hindbrain neural crest is controlled by a combinatorial pattern of *Hox* gene expression [26]. Among the *Hox* family, *Hoxa3* is expressed in the hindbrain neural tube in rhombomeres 5 and 6 [27]. The neural crest cells populating the third pharyngeal arch originate from rhombomeres 5, 6 and 7 [28]. These cardiac neural crest cells express *Hoxa3* as does the endodermal eptithelium of the third pharyngeal pouch [29]. In *Hoxa3* null mutant embryos, the neural crest cell population fails to induce differentiation of surrounding arch and pouch tissues leading to abnormal development of the third arch artery, and defects in the thymus, parathyroid gland and carotid body [30], [31]. Interestingly, a recent study has shown that *Hoxa3* is also expressed in a sub-population of SHF cells that contributes to the OFT myocardium [32].

A classical approach to gain insight into regulatory mechanisms controling *Hox* gene expression is to dissect the flanking regions and test for activity in transgenic embryos. To document the expression pattern provided by the proximal 2 kb of the *Hoxa3* promoter in transgenic mice, we generated *lacZ* transgenic mice that revealed ß-galactosidase expression in specific territories including particular hindbrain, ganglionic and branchial compartments development of which relies on *Hoxa3* activity [33]. In addition to the neural tissue, we observed ß-galactosidase expression in the heart of the transgenic embryos. In this study, we document the expression of this reporter transgene and show that it is specifically expressed in three cell populations important for OFT development. It is first activated in a subpopulation of the SHF expressing *Isl1* and later in OFT myocardial cells. Second, the transgene is expressed in neural crest cells migrating into the 3^rd^, 4^th^ and 6^th^ PAs at E9.5-E10.5. Third the pharyngeal endoderm surrounding the SHF exhibits strong ß-galactosidase expression from E8-8.5 onwards. At E10.5, high level of ß-galactosidase expression is detected in SHF-derived myocardial cells and cardiac neural crest cells that populate OFT cushions, as demonstrated by co-localisation of ß-galactosidase with SHF and cardiac neural crest cells markers. Interestingly, manipulation of the Retinoic Acid (RA) signaling pathway using *Raldh2^-/-^* embryos or injection of all-*trans*-RA reveals that *Hoxa3-lacZ* expression is sensitive to RA dosage. This transgenic line will therefore be of particular use for further investigation of the cellular and molecular interactions between SHF, cardiac neural crest cells and pharyngeal endoderm.

## Results

### The *Hoxa3-lacZ* transgene is active in the pharyngeal region and cardiac neural crest cells

The expression profile of the proximal 2 kb promoter of Hoxa3 was previously examined both in transgenic mouse line and in transient transgenic embryos. This transgene recapitulates a subset of the *Hoxa3* gene expression in specific territories including particular hindbrain, ganglionic and branchial compartments development of which relies on *Hoxa3* activity [33].

**Figure 1:**
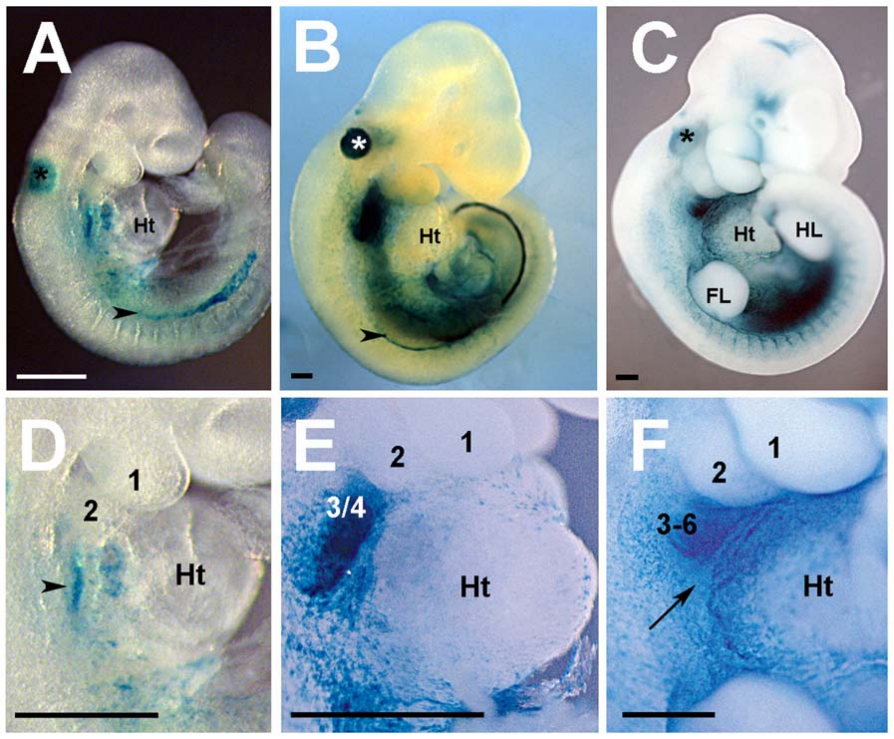
Time course of *Hoxa3-lacZ* transgene activity at early embryonic stages. (A, B, C) Lateral view of E8.5 (A), E9.5 (B) and E10.5 (C) transgenic embryos stained for *lacZ* activity and corresponding magnification of the pharyngeal and heart region, (D, E and F, respectively). Arrowheads in A and B indicate the lateral plate mesoderm; the arrowhead in D shows the pharyngeal endoderm; the arrow in F indicates the pharyngeal mesoderm lying caudally to the OFT. Asterisks in A-to-C indicate the otic vesicle. Numbering 1, 2, 3, 4 and 6 indicates the pharyngeal arches. FL; forelimb; Ht, heart; HL, hindlimb. Scale bar = 100 µm.

After four backcrosses in an outbred, CD-1 mouse genetic background, the transgenic line derived from the construct (H3TG3, 33) exhibited a slightly narrowed expression pattern in whole mount stained embryos with respect to our initial record (Figure 1). At embryonic day (E) 8.5 X-gal staining of *Hoxa3-lacZ* transgenic embryos revealed detectable ß-galactosidase activity at the dorsal midline of the neural tube, in lateral plate mesoderm, in otic vesicles and in the pharyngeal region (Figure 1A). This pattern was similar at later stages including E9.5 (Figure 1B), E10.5 (Figure 1C) and E11.5 (not shown). Focusing on the pharyngeal region, ß-galactosidase activity was confined in the pharyngeal endoderm and developing pharyngeal arches (PAs) at E8.5 (Figure 1D and not shown). By E9.5 and E10.5, whole mount stained embryos exhibited ß-galactosidase activity in PA3, 4 and 6 (Figure 1E, F). Since pharyngeal tissue provides different cellular progenitors crucial for outflow tract (OFT) development, we extended our analysis of *Hoxa3-lacZ* transgene expression during cardiac development.

**Figure 2:**
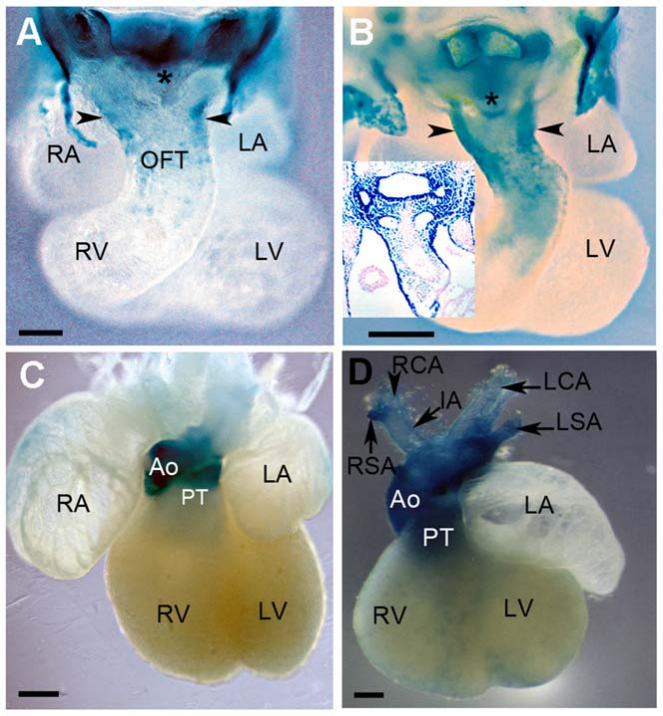
Expression of the *Hoxa3-lacZ* transgene during cardiogenesis. Ventral views of whole mount hearts X-gal stained at E9.5 (A), E10.5 (B), E13.5 (C) and E15.5 (D). (A) X-gal stained cells are present in the inferior and superior walls of the distal OFT (arrowheads), and in the aortic sac indicated by the asterick. (B) ß-gal activity is extended in the proximal OFT and clearly visible in the aortic sac but still excluded from atria and ventricles (arrowheads indicate the transverse section level corresponding to the image inset). (C) Labeled cells are detected in the aortic and pulmonary trunk at E12.5. (D). At E15.5 X-gal staining in the aorta and pulmonary trunk includes the myocardium at the base of these vessels. At all these stages *Hoxa3-lacZ* is silent in the atria and ventricles (A–D). Ao, aorta; IA, innominate artery; LA, left atrium; LCA, left common artery; LSA left subclavian artery, LV, left ventricle; OFT, outflow tract; PT, pulmonary trunk; RA, right atrium; RCA, right common artery; RSA, rigth subclavian artery;RV, right ventricle. Scale bars = 100 µm.

**Figure 3:**
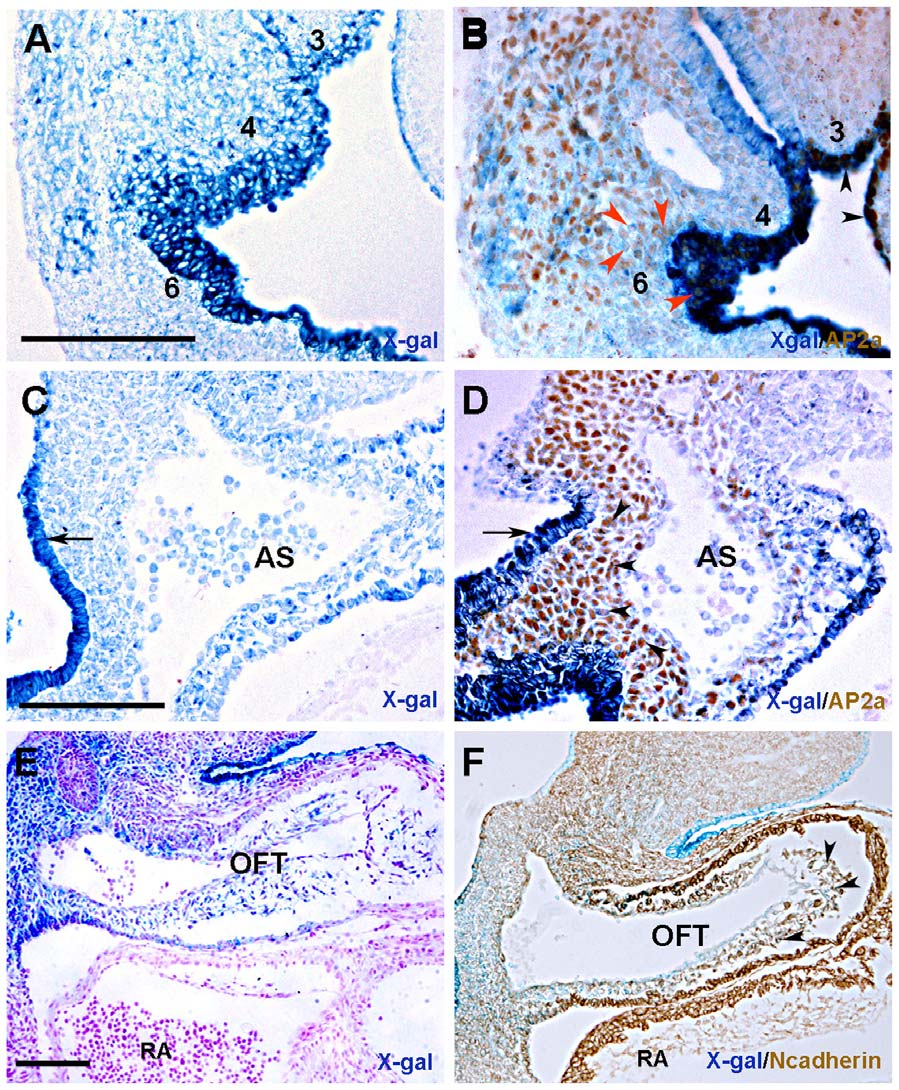
*lacZ*-positive cells in the pharyngeal arches, aortic sac and OFT identify migratory neural crest cells. Sagittal sections of E10.5 transgenic embryos at the level of the 3^rd^, 4^th^ and 6^th^ pharyngeal arches (A, B), aortic sac (C, D), and outflow tract (E, F). Ap2α (B, D) and N-cadherin (F) immunochemistry on X-gal-stained adjacent sections demonstrates that *lacZ*-positive cells in the pharyngeal arches (B) as well as those populating the space between the pharyngeal endoderm and aortic sac (D) and outflow tract mesenchyme (F) are cardiac neural crest cells. Arrowheads and arrows (B) indicate the pharyngeal ectoderm and AP2^+^ LacZ^+^ cells in pharyngeal arches ectoderm and mesoderm, respectively. Arrows and arrowheads (C, D) show the pharyngeal endoderm and AP2^+^ LacZ^+^ cells, respectively. AS, aortic sac; OFT, outflow tract. RA, right atrium 3, 4, and 6 indicate the 3^rd^, 4^th^ and 6^th^ pharyngeal arches, respectively. Scale bars = 100 µm.

We first stained whole transgenic hearts at different developmental stages (Figure 2). ß-galactosidase activity was essentially detected in the arterial pole (outflow tract) of E9.5 (Figure 2A), E10.5 (Figure 2B), E12.5 (Figure 2C) and E15.5 (Figure 2D) transgenic hearts. Since both SHF and cardiac neural crest cells contribute to OFT development during cardiogenesis, we investigated the nature of ß-galactosidase-positive cells in this region. As previously reported, mesenchymal cells in the 3^rd^ and 4^th^ PAs expressed the *Hoxa3-lacZ* reporter at E9.5 [33] and E10.5 (Figure 3). Histological sections from E10.5 transgenic embryos confirmed clear ß-galactosidase expression in PA3, 4 and 6 (Figure 3A) and OFT mesenchyme (Figure 3E). Of note, at E15.5, X-gal labeled cells were found associated with vessels deriving from these three caudal PAs (Figure 2D). These observations suggest that X-gal labeled cells are cardiac neural crest cells. In addition to surface ectoderm, neural crest cells including the cardiac neural crest cell population are a prominent site of AP-2α expression [34]. Therefore, we used AP-2α as a specific marker of the migratory cardiac neural crest cell population that populate PA3 to 6 and the OFT of the heart [34], [35]. Immunostaining for AP-2α on transgenic sections at E9.5 and E10.5 showed that X-gal labeled ectodermal cells expressed AP-2α transcription factor as expected (Figure 3B, arrowheads). In addition, ß-galactosidase–expressing mesenchymal cells of PA3-6 also exhibited nuclear staining for AP-2α (Figure 3B) suggesting that these cells are neural crest-derived cells. At E10.5, the aortic sac (AS) expressed the transgene on whole mount heart (Figure 2B, asterisk), and on histological sections X-gal labeled cells were detected in the pharyngeal endoderm and AS (Figure 3C). AP-2α-expressing cardiac neural crest cells could be detected in the region between the pharyngeal endoderm and the AS, and, in contrast to X-gal-positive cells of the pharyngeal endoderm, ß- galactosidase–expressing cells populating the AS were AP-2α positive (Figure 3D). This pattern matches to that previously reported for independent mouse neural crest lineage markers [16], [35], [36], [37].

Expression of AP-2α is mostly extinguished by E11.5 when the cardiac neural crest cells have migrated into the OFT of the developing heart [35] whereas the AP-2α lineage is detected in the OFT cushion in *AP-2α-Cre; R26R* mouse embryos [37]. Cardiac neural crest cells undergo extensive cell rearrangements during the formation of the aorticopulmonary septum in the OFT which require N-cadherin [38]. We therefore analyzed N-cadherin expression in wild-type and transgenic embryos at E9.5 and E10.5 as an alternative marker of cardiac neural crest cells (Figure S1). At E9.5, cardiac neural crest cells have not yet migrated into the OFT, whereas N-cadherin was already expressed in OFT, atrial and ventricular myocardium (Figure S1A, C). At E10.5, cardiac neural crest cells migrated into the distal end of the OFT, as demonstrated by detection of N-cadherin in this region, although at lower levels compared to myocardial expression (Figure S1B, D) [38]. Immunostaining for N-cadherin on E10.5 transgenic hearts demonstrated that ß-galactosidase activity colocalized with the N-cadherin protein in the OFT mesenchyme (Figure 3E, F), suggesting that these transgenic cells represent cardiac neural crest cells migrating into the OFT. In conclusion, the *Hoxa3-lacZ* transgene was expressed in migratory cardiac neural crest cells of the caudal PAs (3, 4 and 6) and in cardiac neural crest derived mesenchymal cells that populate the cushions in the OFT between E9.5 and E10.5, prior to formation of the aorticopulmonary septum.

**Figure 4:**
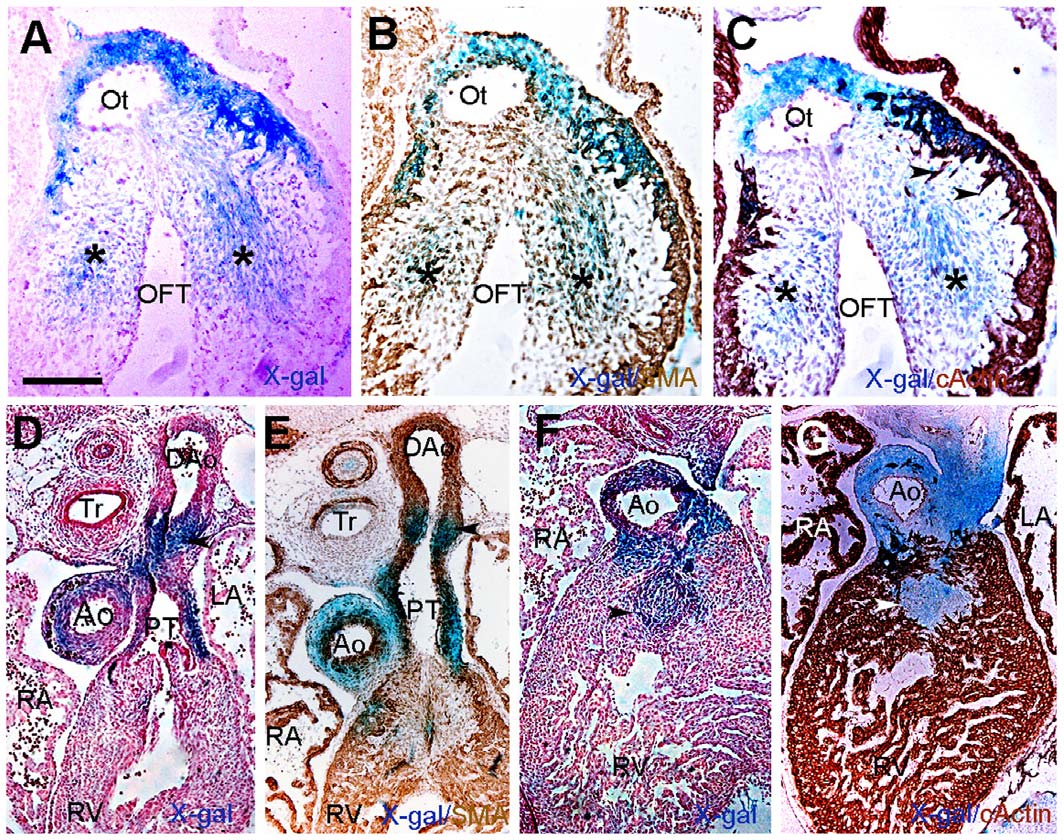
Distribution of X-gal-labeled cells in transgenic hearts before (E11.5) and after (E13.5) outflow tract septation. Transverse sections of E11.5 (A-C), and E13.5 (D-G) developing hearts (A) X-gal-labeled cells are present in the cushion of E11.5 embryos (asterisks) and (B) on adjacent section, α-SMA staining shows that *lacZ*-expressing cells correspond to cardiac neural crest differentiating into smooth muscle that (C) are negative for c-actin expression and are thus not muscularized. Arrowheads in C indicate myocardial spikes protruding toward the cushion mesenchyme. (D) X-gal stained cells are detected in the aortic and pulmonary trunk as well as in the ductus arteriosus (arrowhead). (E) These *lacZ*-positive cells also express α-SMA. (F) At E13.5, the outlet septum stains positive for X-gal (arrowhead). (G) c-actin immunostaining demonstrates that myocardialization occurs in this region as well as in the cushion at base of the aorta. Ao, aorta; DAo, dorsal aorta; LA, left atrium; OFT, outflow tract; Ot, outlet; PT, pulmonary trunk; RA, right atrium; RV, right ventricle; Tr, Trachea. Scale bars = 100 µm.

Since cardiac neural crest cells differentiate into smooth muscle cells (SMC) during formation of the aorticopulmonary septum, we tested whether differentiated SMC were also ß- galactosidase-positive in the cushions (inset Figure 2B). By E11.5, reporter activity was observed in the OFT of whole mount stained hearts (not shown). Histological sections confirmed X-gal labeled cells in the cushions (Figure 4A, asterisks). Immunostaining for smooth muscle α-actin (α-SMA) demonstrated that ß-galactosidase-positive cells in mesenchyme expressed α-SMA (Figure 4B). Indeed, those ß- galactosidase- and α-SMA-positive cells were negative for the marker cardiac actin (c-actin) on adjacent sections (Figure 4C) that is normally expressed by myocardial cells that penetrate the septum from the myocardial wall and are also α-SMA-positive (Figure 4B, C), as previously observed [39]. By E13.5, the aorta and pulmonary trunk contained X-gal labeled cells (Figure 2C). On histological sections, ß- galactosidase-positive cells were detected in the aortic and pulmonary trunks as well as in the ductus arteriosus derived from the left 6^th^ aortic arch artery (Figure 4D) and co-expressed α-SMA on adjacent sections (Figure 4E). At E13.5, the mesenchymal outlet septum formed by fusion of the OFT cushions exhibited ß-galactosidase activity (Figure 4F, 4G, arrowhead). These cardiac neural crest derived mesenchymal cells were previously demonstrated to be derived from the AP-2α lineage [16], [37]. These cushions were in the process of extensive muscularization as revealed by c-actin staining (Figure 4G). Scattered β- galactosidase/c-actin-double positive cells were observed in the aortic trunk but not in the ductus arteriosus where ß-galactosidase-positive cells were not stained for c-actin (Figure 4G). Together, the pattern of transgene expression in the PAs and arterial pole of the heart recapitulated a subset of the cardiac neural crest migratory route [16], [18], [35], [37].

### Expression of the transgene in a subdomain of the SHF and OFT myocardium

**Figure 5:**
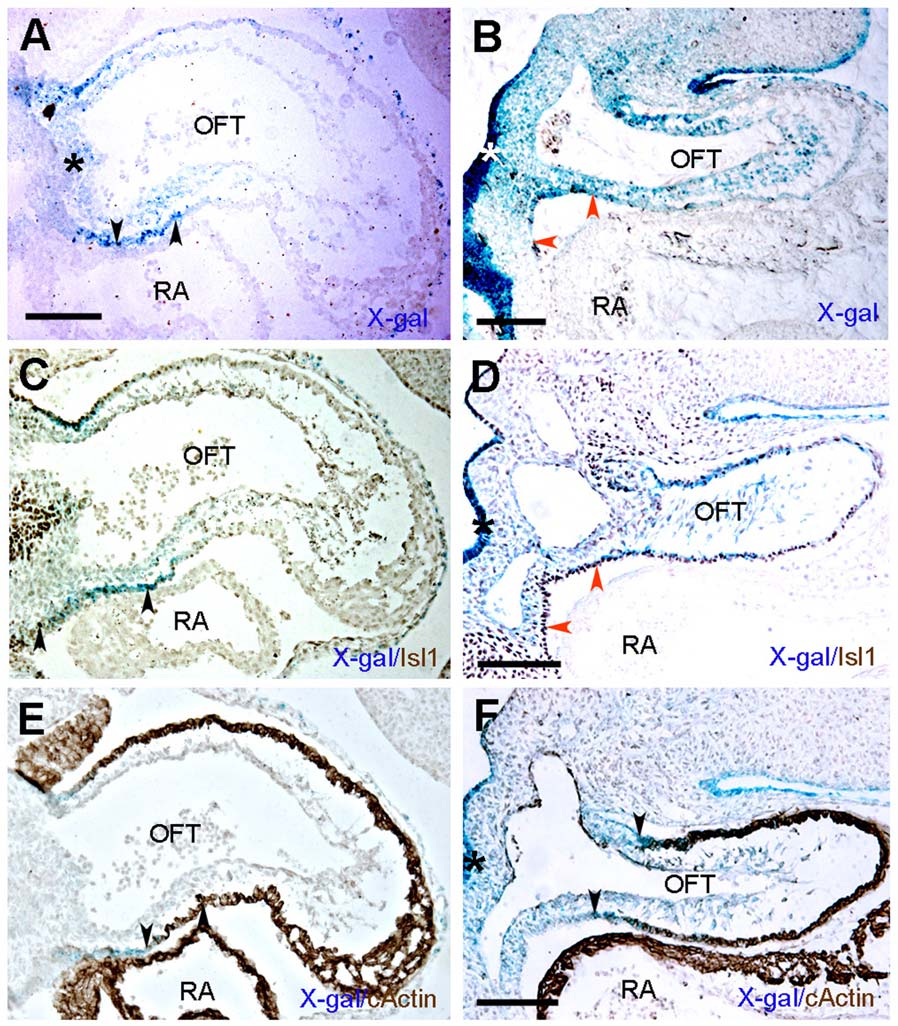
β-gal, Islet-1 and cardiac actin expression in the hearts of transgenic embryos at E9.5 and E10.5. Transverse (A, C, E) and sagittal (B, D, F) sections of E9.5 and E10.5 transgenic embryos, respectively. (A) The X-gal-stained cells in the outer layer of the outflow tract also express Isl1 (C, arrowheads). Only transgenic cells in the distal outflow tract (delineated by arrowheads in A) express c-actin (E, arrowheads). Note that dorsal part of the developing aortic sac (asterisk in A) is filled with Isl1-expressing cells. (B) X-gal-stained cells are present in the outflow tract region and anterior portion of SHF demarcated by the red arrowheads. (D) Isl1-immunostained cells are present in this anterior portion of SHF (demarcated by the red arrowheads) and outflow myocardium, whereas c-actin is restricted to myocardial transgenic cells of the outflow tract (F, arrowheads). OFT, outflow tract. Black and white asterisks in (A) and (B) show the aortic sac and pharyngeal endoderm, respectively. Scale bars = 100 µm.

SHF progenitor cells are characterized by the expression of *Isl1* [5], *Nkx2.5* [9], *Fgf8*, *Fgf10* [4], and *Tbx1* [11], and cells that have transcribed these genes form the arterial pole of the heart. The *Mef2C SHF* enhancer (*Mef2c-AHF-lacZ*) [40] and the *Mlc1v-nlacZ-24* transgene [4] are also expressed in the SHF. Cell tracing and explant culture experiments have shown that SHF progenitor cells contribute to the inflow and outflow tracts [4], [5], [6], [7]. As mentioned above, *Hoxa3-lacZ* transgenic embryos exhibited X-gal staining in the forming posterior PAs and pharyngeal endoderm at E8.5 (Figure 1D, arrowhead). At E9.5, on whole mount stained heart, transgene expression was detected in the distal region of the OFT (Figure 2A). Histological sections showed that X-gal-labeled cells were restricted to a subdomain of the SHF, while the overlying pharyngeal endoderm cells displayed broad X-gal staining (Figure S1G). Between E10 and E10.5, we observed increased X-gal staining in the OFT (Figure 2B) as well as in pharyngeal mesoderm caudal to this region (Figure 1F, arrow). Sections confirmed reporter activity in OFT myocardium (Figure 5A, B) as well as in a sub-region of the SHF (Figure 5B). To better characterize the identity of the X-gal labeled cells in the SHF and OFT myocardium, we performed immunostaining for Isl1 protein and c-actin, as a marker of differentiated cardiomyocytes, in E9.5 and E10.5 transgenic embryos. Isl1 protein is used as a pan-marker of SHF cells as well as cardiomyocytes of the distal OFT [9], [41]. Results showed that X-gal labeled cells were either single positive for Isl1 or double Isl1/c-actin positive (Figure 5C-F, and Figure S1H). Taken together, these results showed that the transgene is expressed in the anterior SHF, in differentiating as well as recently differentiated cardiomyocytes of the distal portion of the OFT. The changing position of X-gal labeled cells during OFT formation therefore provided a “chase”, marking cells which previously transcribed the *Hoxa3-lacZ* transgene. Similarly, in *Mef2C-AHF-lacZ* [40] and *Mlc1v-nlacZ-24* [4] transgenic embryos, ß-galactosidase expression were more extended in the SHF and included the OFT and right ventricle at E8.5 and E9.5 (see below, Discussion). In accordance with restricted staining of the SHF by *Hoxa3-lacZ* cells, the inflow tract, atria and right ventricle were negative for ß-galactosidase activity between E9.5 and E15.5.

Therefore, at E10.5, the *Hoxa3-lacZ* transgene is activated in both cardiac neural crest and SHF-derived myocardial cells at a crucial time of OFT development.

### *Hoxa3- lacZ* activity is sensitive to Retinoic Acid signaling

Retinoic Acid (RA) is the active derivative of vitamin A that plays crucial roles in various steps of cardiovascular development [42], [43]. In addition, a recent study has shown that expression of *Hoxa3* in a sub-population of SHF cells depends on RA signaling [32]. We therefore analyzed the responsiveness of *Hoxa3-lacZ* transgene to changes in RA signaling in transgenic embryos.

**Figure 6:**
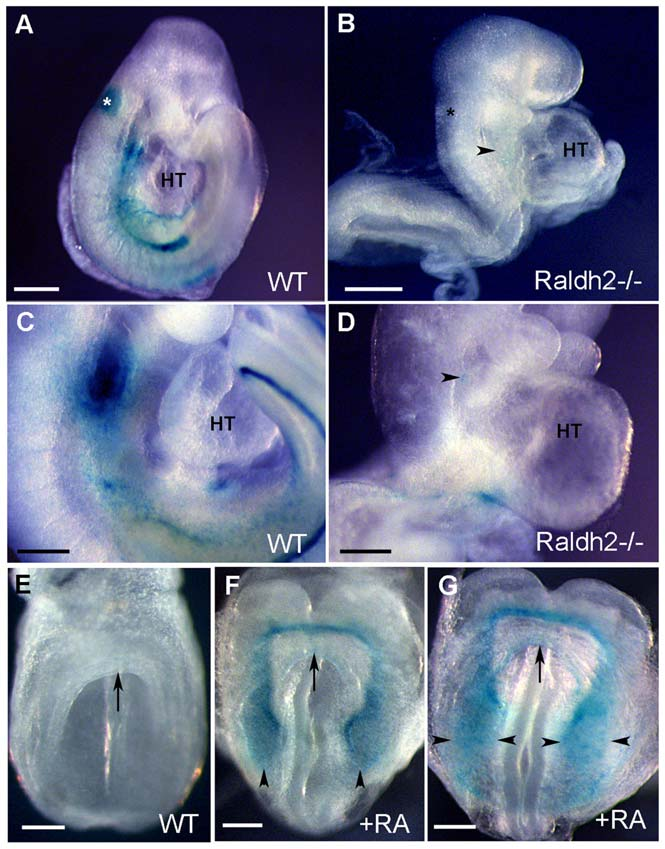
RA signaling affects *Hoxa3-lacZ* expression in transgenic embryos. Lateral views of E8.5 (A, B), E9.5 (C, D), and frontal views of E8 (E,F,G) embryos. In contrast to control *Hoxa3-lacZ* embryos (A, C), ß-galactosidase activity is lost in *Hoxa3-lacZ*/ *Raldh2−/−* embryos (B, D). *Hoxa3–lacZ* embryos treated with vehicle (DMSO) show no activation of the transgene (E) whereas RA-treated transgenic embryos display a precocious ß-galactosidase upregulation in the SHF (F, G, arrowheads). Arrows (F, G) indicate the cardiac crescent. Asterisk indicates the otic vesicle. HT, heart tube. Scale bars = 100 µm.

To visualize the *Hoxa3-lacZ* expression pattern in the absence of RA signaling, we generated embryos harboring the *Hoxa3-lacZ* transgene but deficient for retinaldehyde dehydrogenase 2 (*Raldh2^−/−^)*, the enzyme that catalyzes the second oxidative step in RA biosynthesis [44]. As previously reported, *Raldh2^−/−^* embryos failed to undergo heart looping and have impaired atrial and sinus venous development compared to wild-type hearts [45] (Figure 6A, B). In contrast to wild-type *Hoxa3-lacZ* transgenics, *Raldh2^−/−^;Hoxa3-lacZ* embryos lacked ß-galactosidase activity in the entire embryo except for faint staining in the pharyngeal region at E8.5 (compare Figure 6A and 6B) and E9.5 (Figure 6C, D). *Hoxa3-lacZ* expression was thus abrogated under RA deficiency showing that transgene-expressing cells are regulated by RA signaling.

Because cardiac patterning is sensitive to administration of exogenous RA at early stage (E6.75–E8.5) [46], [47], [48], [49], we have also examined the effect of exogenous RA on transgene expression in the embryo at a stage when cardiac neural crest and SHF cells are responsive to RA [46], [47], [48], [49]. In contrast to *Hoxa3-lacZ* control E7.5 embryos treated with vehicle only (Figure 6E), those treated with exogenous RA exhibited an up-regulation of *Hoxa3-lacZ* in the heart field at E8 (Figure 6F, G), confirming that the transgene is sensitive to an increase in RA dosage.

## Discussion

In this study, we characterize transgenic embryos harboring a reporter transgene controlled by the proximal 2 kb of the *Hoxa3* gene promoter that is expressed in several tissues required for OFT development and depends on the RA signaling pathway. Using X-gal staining and immunostaining for markers of distinct cardiac lineages, we demonstrated that the transgene exhibited β-galactosidase activity in the PAs and the OFT during cardiogenesis. General feature of our analysis may be relevant to the tripartite interactions between cardiac neural crest, SHF and pharyngeal endodermal cells during cardiac development in general and OFT morphogenesis in particular. First, the transgene is expressed in the pharyngeal endoderm from E8.5 to E11.5. Second, the transgene is activated in the migratory cardiac neural crest cells of the PAs and those that populate the OFT and participate in its septation. Third, a sub-domain of SHF progenitor cells expressed the transgene at E9.5 and, most importantly, at E10.5, SHF-derived cells in the OFT further displayed transgene expression. Finally, we observed that transgene activity is lost in *Raldh2^−/−^* mutant embryos while it is precociously and highly up-regulated in all-*trans*-RA treated embryos.

### *Hoxa3-lacZ* expression in cardiac neural crest cells, SHF and OFT myocardium

Our study showed *Hoxa3-lacZ* expression in migratory cardiac neural crest cells that invade the 3^rd^, 4^th^ and 6^th^ PAs [28], OFT, smooth muscle cells of the cushions and aortic and pulmonary trunk as well as in those contributing to the developing 3^rd^, 4^th^ and 6^th^ aortic arch arteries. In all these territories, ß-galactosidase activity colocalised with cardiac neural crest markers such as AP-2α and N-cadherin. Interestingly, the expression pattern of *Hoxa3-lacZ* in the cardiovascular system partially recapitulated that previously reported for transgenic lines expressing *lacZ* in cardiac neural crest under the control of the proximal 650-bp of the *Connexin43* gene promoter [18] as well as of those obtained upon Cre/lox-mediated induction involving the neural crest specific *Pax3, Wnt1* or human tissue plasminogen activator (Ht-PA) promoters [16], [17], [18], [50]. Our results are consistent with previous studies showing that *Hoxa3* expression is detected in the neural crest cells migrating into the 3^rd^, 4^th^ and 6^th^ PAs [29], [51]. Moreover, in *Hoxa3* mutant embryos the third arch arteries degenerate [30], [31].

**Figure 7:**
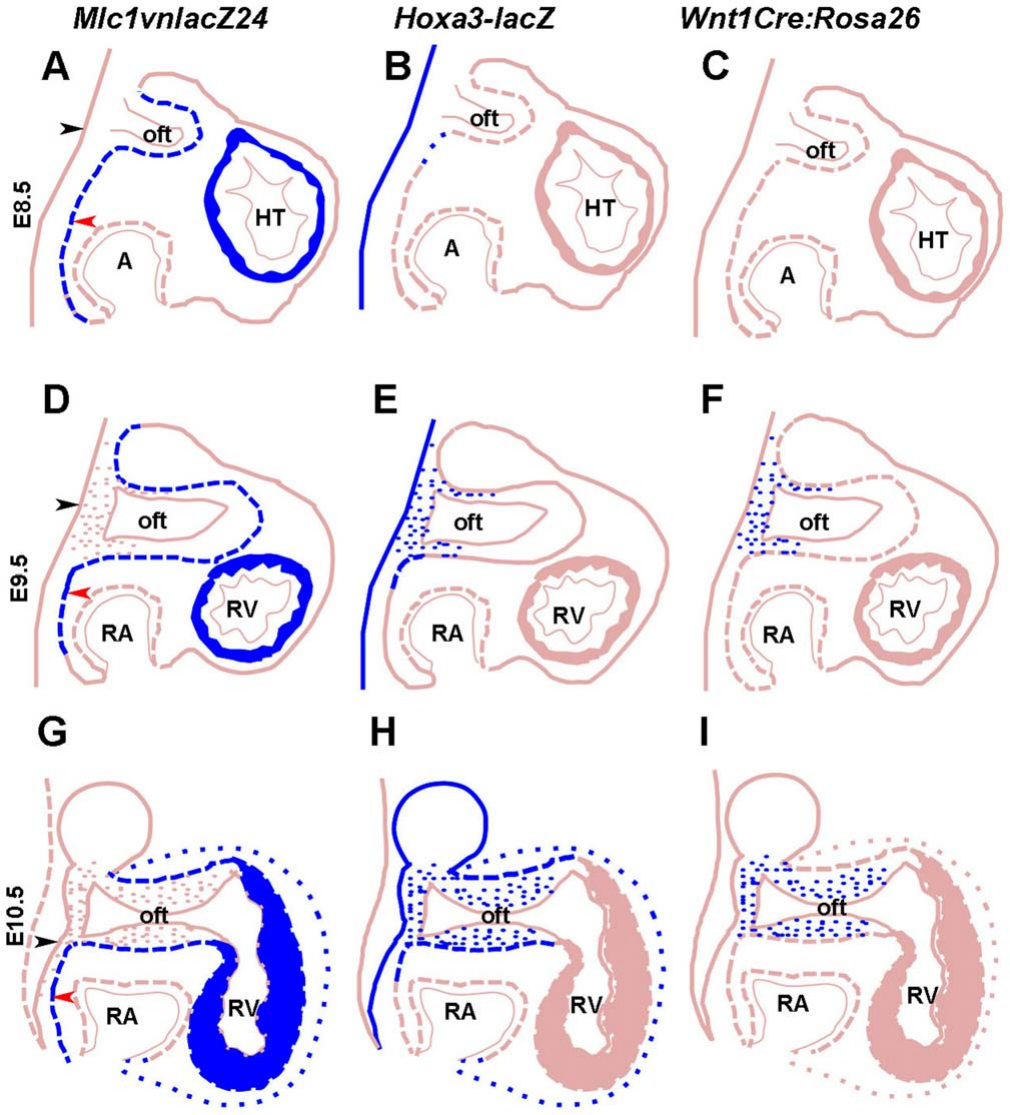
Schematic view of *lacZ* expression pattern in of *Mlc1v-nlacZ24, Hoxa3-lacZ* and *Wnt1Cre:R26R* cardiosensor lines. *Mlc1v-nlacZ24* (A, D, G), *Hoxa3-lacZ* (B, E, H) and Wnt1*Cre:R26R* (C, F, I). At E8.5 *Mlc1v-nlacZ-24* are activated in the second heart field, developing OFT and heart tube (A), while *Hoxa3-lacZ* is activated in PE and barely detected in the SHF (B). At this stage the heart is devoid of migrating cardiac neural crest cells (C). At E9.5 the SHF progenitors, OFT and RV myocardium are labeled in *Mlc1v-nlacZ-24* (D). The PE, CNC (dots in the caudal pharynx) and a subdomain of the SHF express *Hoxa3-lacZ* (E). Cardiac neural crest cells are also labeled in *Wnt1cre;R26R* bigenic embryos at E9.5 (F). At E10.5, *Mlc1v-nlacZ-24* transgenic hearts identify SHF, OFT and RV (G). In addition to the anterior portion of SHF and PE, *Hoxa3-lacZ* transgene activity identifies cardiac neural crest and myocardial cells of the OFT, whereas the RV remains negative for *lacZ* staining (E). Cardiac neural crest cells similarly invades the OFT in *Wnt1cre;R26R* bigenic embryos at E10.5 (I). *Hoxa3-lacZ* clearly appears as an intermediary reporter line that display hybrid expression pattern reminiscent to that of Mlc1v-nlacZ24 and *Wnt1cre;R26R* bigenic. Blue and pink colors indicate *lacZ*-positive and -negative cells, respectively. Black and red arrowheads designate the pharyngeal endoderm (PE) and second heart field (SHF), respectively. A, atria; HT, heart tube; Oft, outflow tract; RA, right atria; RV, rigth ventricle.

In addition to being expressed in the cardiac neural crest that populate the OFT, the *Hoxa3-lacZ* reporter is also active in a sub-domain of the SHF and myocardial cells of the OFT. This expression pattern in SHF progenitors and SHF derived-myocardial cells is in sharp contrast with other previously described SHF reporter lines [4], [5], [40]. Indeed, the *Mlc1v-nlacZ-24* and *Mef2C-AHF-lacZ* reporter transgenes both mark a larger portion of the SHF, and subsequently identify cells that are found in both OFT and right ventricular myocardium [4], [7], [40]. The labeling of these progenitor cells occurred essentially at the time (i.e. E7.5) of anatomic appearance of the SHF [4], [40]. Conversely, *Hoxa3-lacZ* expression was delayed by 24h since *Hoxa3-lacZ* positive cells were detectable by E8.5 and only in a sub-domain of the SHF. Thus, it only partially overlaps with that of the *Mef2C-AHF-lacZ* or *Mlc1v-nlacZ-24* lines (Figure 7). However by E10.5, *Hoxa3-lacZ* transgene expression was extended in the SHF and OFT myocardium, overlapping with *Mef2C-AHF-lacZ* and *Mlc1v-nlacZ-24* transgene activities, except for its exclusion from the right ventricle (Figure 7). Thus, in our transgenic model, the initial SHF progenitors deployed to the heart tube at both poles to generate myocardial cells of the atria and right ventricle are negative for *Hoxa3-lacZ* (Figure 7). Subsequently (i.e. at E10-10.5) the entire OFT myocardium (also labeled by *Mlc1v-nlacZ-24* and *Mefc2C-AHF-lacZ*) expresses *Hoxa3-lacZ* concurrently with cardiac neural crest cells populating the OFT. This cardiac neural crest contribution would bring cells needed in the OFT to interact with the underlying myocardium for correct OFT development at E9.5–10.5 [16]. It has been reported that the presence of cardiac neural crest cells in the caudal pharynx is crucial for the addition of SHF to the OFT myocardium [19], [20], [52], [53], but to date, no gene and/or reporter transgene were reported to be expressed in the underlying pharyngeal endoderm, cardiac neural crest cells and SHF–derived myocardium during OFT development. For the first time here, we describe a transgenic line (*Hoxa3-lacZ*) which therefore appears attractive to investigate potential “tripartite” interactions between these cell populations crucial for OFT development.

The *Mlc1v-nlacZ-24* and *Mef2C-AHF-lacZ* transgenes show *bona fide* expression patterns with respect to the *Fgf10* and *Mef2C* enhancer elements from which they are derived respectively [4], [40]. A recent study shows *Hoxa3* expression in a sub-population of SHF cells that contribute to OFT myocardium [32]. Thus, the *Hoxa3-lacZ* transgene is likely to reflect a specific activity of *Hoxa3* in SHF cells.

### *Hoxa3-lacZ* and RA signaling

Several lines of evidence show that the expression of some *Hox* genes from paralogous group 1 is dependent on RA signaling [54], [55]. Moreover, the study by Bertrand et al. (2011)[32] shows that *Hoxa3* expression in the SHF is dependent on RA signaling. Here, we showed that RA deficiency negatively affected the *Hoxa3-lacZ* in all the territories in which it is active including the PAs, OFT and pharyngeal endoderm whereas exogenous RA induced its precocious activation and further enhanced its activity in cardiac progenitors. These data therefore demonstrate that *Hoxa3-lacZ* is sensitive to RA dosage. The *Hoxa3-lacZ* transgene was made up of the proximal 2 kb of the *Hoxa3* gene promoter [33]. Careful sequence analysis allowed predicting a highly conserved DR5-RARE element, which could mediate this RA responsiveness (Figure S2). Alternatively, it may be possible that this *Hoxa3* promoter region is indirectly regulated by RA signaling via an RA sensitive *Hoxa3* transcriptional modulator.

RA deficiency alters gene expression in the SHF and induces a posterior expansion of markers of the SHF, including *Tbx1*, *Fgf8*, *Islet1* and the *Mlc1v-nlacZ-24* reporter transgene [48], [49]. This is in sharp contrast with the results reported here for *Hoxa3-lacZ*;*Raldh2^-/-^* embryos. However, the sensitivity of the transgene to RA is consistent with the down regulation of *Hoxa3* reported in the hindbrain and pharyngeal region of *Raldh2^−/−^* embryos [56]. A recent report on the fate of RA–activated embryonic cell lineages revealed that among many tissues, migratory cardiac neural crest cells of PA3 to 6, pharyngeal endoderm, atrial chamber, OFT, aortic arches, and dorsal aorta are responsive to RA signaling [57]. Hence, the loss of *Hoxa3-lacZ* transgene expression in *Raldh2^−/−^* embryos at E8.5 and E9.5 may result from cell death and/or failure in the development of cardiac neural crest cells fated to populate PA 3, 4 and 6. Indeed, increased apoptosis was observed in the hindbrain and neural crest cells of *Raldh2^−/−^* embryos [56], and a loss of X-gal staining in these tissues was reported at E8.5 and E9 in *Raldh2^−/−^* embryos harbouring *RARE*-*Cre* and *R26R* transgenes [57].

*Hoxa3* expression has been reported to be altered in RA-exposed embryos [58], [59]. Indeed, RA-exposed embryos exhibited precocious and increased *Hoxa3* expression within its normal expression domain and expression in the hindbrain was shifted rostrally. Among the developmental defects reported in these RA-treated fetuses, transposition of the great arteries or stenosis of the carotid artery, interrupted aortic arch, double outlet right ventricle, truncus arteriosus with a high ventral septal defect and insertion of the internal and external left carotid arteries directly in the aortic arch, were reported [58]. These defects are reminiscent of those observed in several genetic models of congenital heart diseases affecting SHF progenitors and cardiac neural crest cell development [22], [23]. The precocious or ectopic activation of *Hoxa3* in RA-treated embryos may reflect the way changes in the combinatorial *Hox* code affect cardiac development [32], [58], [60], [61]. RA may thus act as a global morphogen involved in coordinated *Hox* gene expression in cardiac progenitor cells, as it does for embryo axial structures.

### Conclusion

We have described a mouse reporter transgene which delineates three crucial cell lineages for the OFT development. In contrast to the cardiosensor lines reported until now, this *Hoxa3-lacZ* reporter is the first that is expressed in cardiac neural crest cells, SHF-derived OFT myocardium and pharyngeal endoderm at the period of OFT morphogenesis. The other reported lines mark either cardiac neural crest cells [16], [17], [18], [50], [62] or the OFT myocardium [4], [40] but not both cell populations. This line appears as an alternative tool for further studies of interactions between cardiac neural crest cells, OFT myocardial cells and pharyngeal endoderm in specific genetic systems and for understanding the etiology of congenital heart defect.

## Materials and Methods

### Ethics statement

All animal work has been conducted according to relevant national and international guidelines and approved by the “Comité d'éthique pour l'expérimentation animale, Université catholique de Louvain, Louvain-la-Neuve” (approval number # 053001).

### Mouse lines

The reporter transgene we generated, referred to as *Hoxa3-lacZ*, consists of the *Escherichia coli lacZ* gene fused to a 2-kb fragment of the *Hoxa3* promoter extending up to the ATG codon of the *Hoxa3* reading frame (Figure S2). The detailed transgene construction and mouse transgenesis have been described previously [33].

The *Raldh2* mutant line was described by Niederreither et al. (1999)[44]. To assess the expression *Hoxa3-lacZ* in Retinoic Acid deficient background, males hemizygote for *Hoxa3-lacZ* and heterozygote for *Raldh2 (Hoxa3-lacZ; Raldh2^−/+^)* were crossed with heterozygote female *Raldh2^−/+^* to generate E8.5 and E9.5 *Hoxa3-lacZ; Raldh2^−/−^* embryos.

### X-gal staining and immunohistochemistry

To document the expression pattern of the reporter, embryos from E7.5 until E17.5 were analysed both morphologically (whole mount stained) and histologically. At least three transgenic embryos of each stage were analysed according to Theiler's nomenclature. For *lacZ* staining, embryos obtained between E7.5 and E17.5 were fixed in 4% paraformaldehyde for 30 to 90 min at 4°C. They were then washed twice in 1% phosphate buffer saline (PBS) at room temperature for 20 min and stained overnight at 30°C in 40 mg/ml X-gal, 200 mM K_3_Fe(CN)_6_, 200 mM K_4_Fe(CN)_6_, 1M MgCl_2_, 1xPBS. For histological analyses, embryos to be sectioned were fixed overnight in 4% paraformaldehyde at 4°C, stained as above, dehydrated and embedded in paraffin (or stored in 70% ethanol until use). 6–to-8 µm thick sections were counterstained with eosin.

For immunohistochemistry, sections from E9.5–13.5 dissected embryos were deparafinized in xylene, rehydrated and finally washed in 1xPBS. Following antigen retrieval in 10 mM citric acid (pH 6.0), sections were blocked with 5% goat serum in 1xPBS for 1 hour at room temperature and then incubated with various primary antibodies. Endogenous peroxidases samples were inhibited with 3% H2O2 in water for 10–15 min and washed in 1xPBS. For the detection of cardiac actin (c-actin), Islet-1, N-cadherin, AP-2α, PECAM-1 and α-smooth muscle actin (α-SMA) expression, the following antibodies were diluted in a solution of 0.1% Triton X-100/5% goat serum/PBS and used at 4°C O/N at the dilutions shown: anti-mouse c-actin (1:400, A2547 Sigma), anti-rat N-cadherin (1:400, DSHB clone MND2) and anti-mouse Isl1 (1:100, DSHB, clone 39.4D5), AP-2α (1/100, DSHB clone 3B5) and mouse anti α-SMA (clone 1A sigma, 1/1000). The primary antibodies were detected with biotinylated second antibody goat anti-rat, goat anti-mouse, or goat-anti-rabbit (1:200, Vector Laboratories) followed by avidin-biotin binding (Vectastatin ABC kit, Vector Laboratories) and application of 3,3′-diaminobenzidene (Vector Laboratories). After desired color intensity was achieved, reaction was stopped by extensive wash in distilled water. The samples were then dehydrated through increasing ethanol (70%, 95% and 100%), immersed in xylene and mounted in DEPEX (EMS).

For immunofluorescence, sections were treated with sodium borohydride (1 mg/ml) on ice to eliminate the epifluorescence due to fixative, and blocked in a solution of 0.1% Triton X-100/5% donkey serum/1xPBS. The following antibodies were used at the dilutions shown: c-actin (mouse, 1:200, A2547 Sigma), anti-Islet-1 (mouse, 1: 100, DSHB), anti-N-cadherin, (rat, 1:50, DSHB). Following overnight incubation at 4°C and washes in 1xPBS, fluorescently labeled second antibodies (Molecular Probes) were used at a 1:250 dilution to detect primary antibodies: 555-conjugated donkey anti-mouse for c-actin, Alexa 488-conjugated donkey anti-rat for N-cadherin. Sections were washed in PBS, mounted in Prolong with DAPI (Invitrogen) and imaged on a fluorescent microscope for analysis.

### Retinoic Acid Treatment of Embryos

Retinoic Acid treatment of embryos was done as previously described by Ryckebusch et al. (2008)[48]. All-trans-RA (Sigma) was dissolved in DMSO and diluted at 20 mg/ml. At E7.5, the *Hoxa3-lacZ* transgenic mice were given a single intra peritoneal injection of the RA solution (65 mg/kg) or control DMSO. Control and treated embryos were harvested at E7.5, E8 and E8.5 and stained for β-galactosidase activity as described above.

## Supporting Information

Figure S1 **Cardiac expression of N-cadherin, cardiac Actin and Islet-1 at E9.5 and E10.5.** Expression of N-cadherin in the heart of E9.5 (A) and 10.5 (B) transgenic embryos, with corresponding magnification of the outflow tract region (C and D, respectively). Cardiac neural crest cells lack N-cadherin expression at E9.5 (arrowhead in C), while it is upregulated at E10.5 (arrowheads in D). (E) At E9.5, cardiac actin is excluded from SHF progenitor cells (arrowheads). Arrowhead in (F) indicates the posterior limit of differentiated cells positive for c-actin in the outflow tract at E10.5. (G) *lacZ* expressing cells are present in the pharyngeal endoderm (arrow) and in a region that expresses Isl1 (H, arrowheads) in addition to the pharyngeal endoderm and outflow tract proper at E9.5. OFT, outflow tract; PE, pharyngeal endoderm; RA, right atria; RV, rigth ventricle. Scale bar = 100 µm.
(TIF)

Figure S2 **Schematic representation of the proximal 2-kb promoter of** ***Hoxa3*** **used to generate the** ***Hoxa3-lacZ*** **reporter transgene.** The Asp718-NruI 2-kb genomic fragment from the mouse *Hoxa3* locus was fused to the *E. coli lacZ* coding sequence to generate the reporter transgene. The sequence of the 5′ moiety of the promoter is shown and contains a predicted DR5 RARE site (underlined bold italics). Asp718 and XbaI restriction sites are shown in bold case.
(TIF)
