# Supplementary figures and images for: A Retinoic Acid Responsive Hoxa3 Transgene Expressed in Embryonic Pharyngeal Endoderm, Cardiac Neural Crest and a Subdomain of the Second Heart Field
Source: PLoS One. 2011 Nov 16;6(11):e27624. doi: 10.1371/journal.pone.0027624 (PMC3217993; doi:10.1371/journal.pone.0027624)

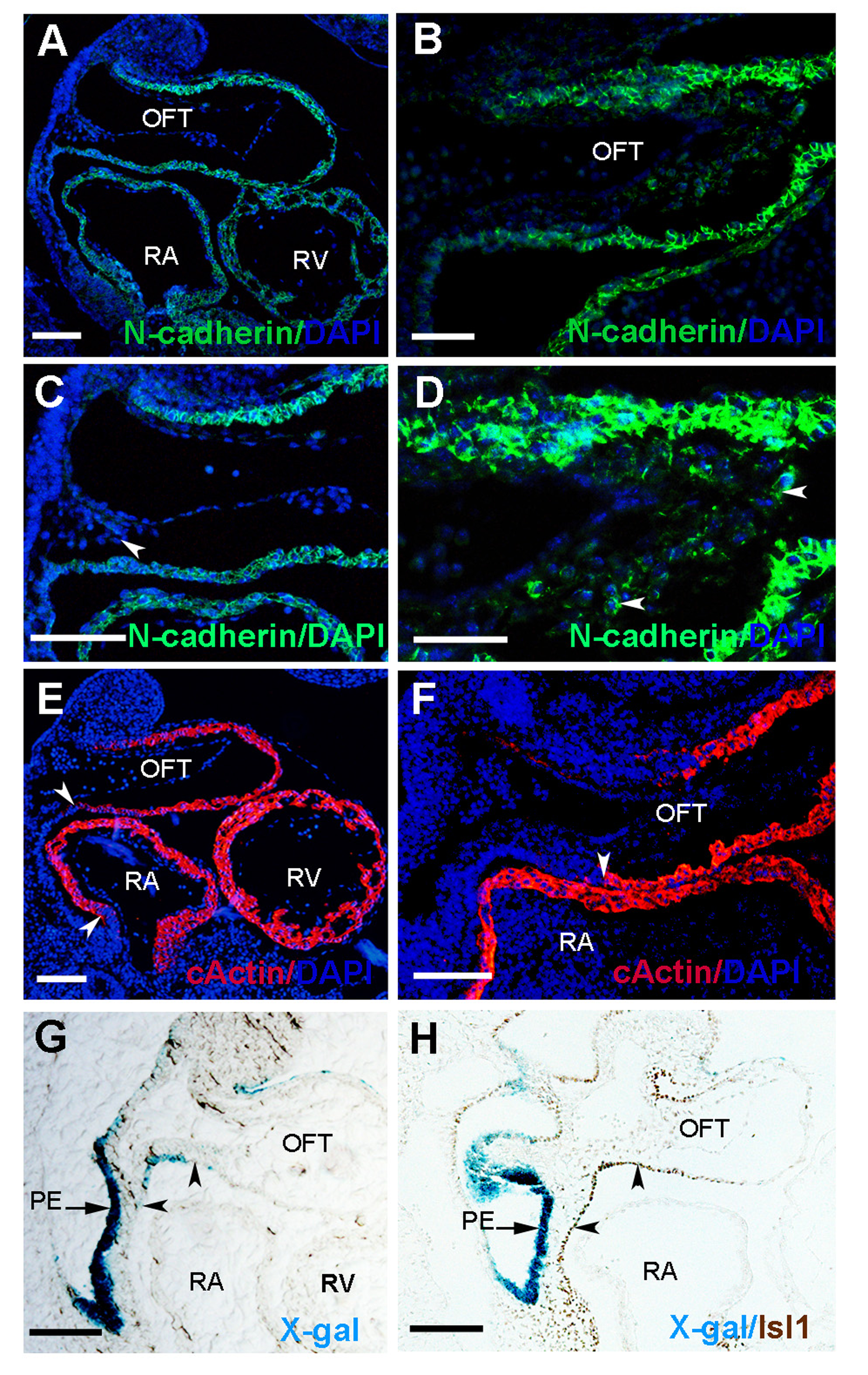

Supplement: Figure S1 — Cardiac expression of N-cadherin, cardiac Actin and Islet-1 at E9.5 and E10.5. Expression of N-cadherin in the heart of E9.5 (A) and 10.5 (B) transgenic embryos, with corresponding magnification of the outflow tract region (C and D, respectively). Cardiac neural crest cells lack N-cadherin expression at E9.5 (arrowhead in C), while it is upregulated at E10.5 (arrowheads in D). (E) At E9.5, cardiac actin is excluded from SHF progenitor cells (arrowheads). Arrowhead in (F) indicates the posterior limit of differentiated cells positive for c-actin in the outflow tract at E10.5. (G) lacZ expressing cells are present in the pharyngeal endoderm (arrow) and in a region that expresses Isl1 (H, arrowheads) in addition to the pharyngeal endoderm and outflow tract proper at E9.5. OFT, outflow tract; PE, pharyngeal endoderm; RA, right atria; RV, rigth ventricle. Scale bar = 100 µm. (TIF) [file pone.0027624.s001.tif]

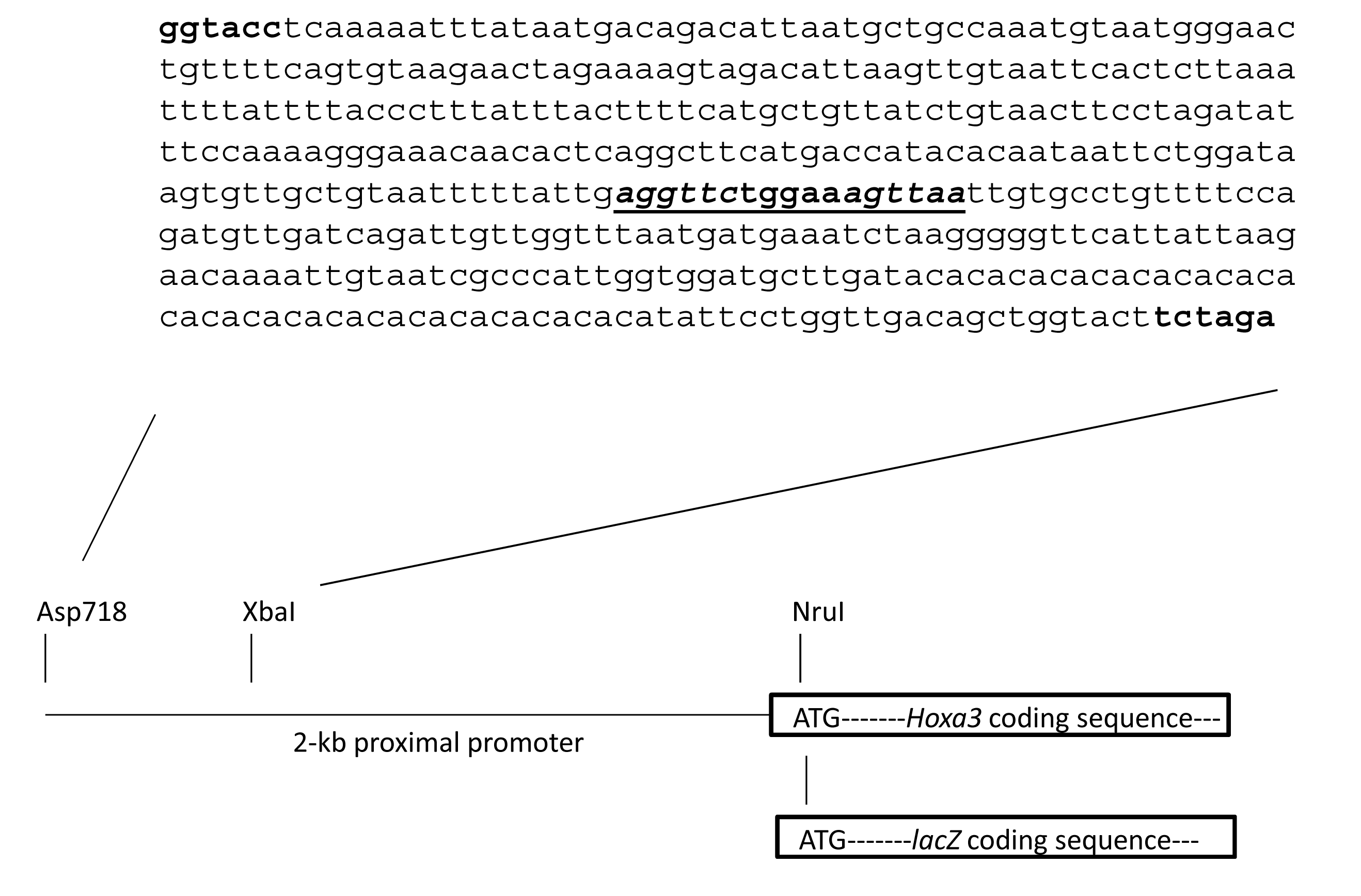

Supplement: Figure S2 — Schematic representation of the proximal 2-kb promoter of Hoxa3 used to generate the Hoxa3-lacZ reporter transgene. The Asp718-NruI 2-kb genomic fragment from the mouse Hoxa3 locus was fused to the E. coli lacZ coding sequence to generate the reporter transgene. The sequence of the 5′ moiety of the promoter is shown and contains a predicted DR5 RARE site (underlined bold italics). Asp718 and XbaI restriction sites are shown in bold case. (TIF) [file pone.0027624.s002.tif]
